# Drought dynamics explain once in a century yellow fever virus outbreak in Brazil with implications for climate change

**DOI:** 10.1126/sciadv.adz6832

**Published:** 2026-08-14

**Authors:** Jamie M. Caldwell, Bryan Grenfell, Gabriel Vecchi, Joelle I. Rosser

**Affiliations:** ^1^ High Meadows Environmental Institute, Princeton University, Princeton, NJ 08540, USA.; ^2^ Department of Ecology and Evolutionary Biology, Princeton University, Princeton, NJ 08540, USA.; ^3^ Department of Geosciences, Princeton University, Princeton, NJ 08540, USA.; ^4^ Stanfor University School of Medicine, Stanford University, Stanford, CA 94305, USA.

## Abstract

While excess rainfall is associated with mosquito-borne disease because it supports mosquito breeding, drought may also counterintuitively increase disease transmission by altering mosquito and host behavior. This phenomenon is important to understand because climate change is projected to increase both extreme rainfall and drought. In this study, we investigated the extent to which seasonally driven mosquito and primate behavior drove the first yellow fever virus (YFV) epidemic in an urban area in Brazil in nearly a century, coinciding with an equally rare drought, and assessed the role of interventions in ending the outbreak. We hypothesized that drought-like conditions played a key role in the outbreak dynamics by driving the forest mosquitoes and nonhuman primates toward the city in search of water and that the mosquitoes were biting more frequently to avoid desiccation. A dynamical YFV model supports these hypotheses, showing that both behavioral changes were needed to explain the outbreak timing and incidence. Furthermore, a combination of vector control, conservation measures, and vaccination contributed to ending the outbreak, with the strongest effects from vaccination. Together, these results suggest that drought, likely to become more frequent in this region in the coming decades, can considerably influence mosquito-borne disease transmission and that sustained control will require multiple interventions.

## INTRODUCTION

After nearly a century without urban yellow fever virus (YFV) transmission, Brazil experienced a major outbreak in 2017–2018 (*1*, *2*), likely driven by a combination of land use change, viral evolution, accumulation of susceptible populations, and favorable climate conditions. YFV causes disease in humans and nonhuman primates with clinical presentations ranging from asymptomatic to life-threatening. YFV spreads via an urban transmission cycle involving *Aedes aegypti* mosquitoes and humans or a sylvatic (i.e., forest) cycle between forest mosquitoes, nonhuman primates, and occasionally, humans. By the 1930s, aggressive vector control and vaccination had controlled the urban cycle (*3*, *4*), with ongoing YFV transmission in Brazil occurring exclusively through the sylvatic cycle. In recent years, however, intensive land use change facilitated increased transmission of YFV into rural human populations at the forest edge (*5*). As deforestation increased, YFV spread southeastward in Brazil, leading up to the 2017–2018 outbreak (*6*), which was first reported in Minas Gerais and then in neighboring states of Espiritu Santo, Rio, and Sao Paulo (*1*, *7*). The virus may have evolved increased transmutability during this time through phenotypic changes in nonstructural proteins (*8*), and historically low vaccination rates due to low YFV risk (*6*, *9*, *10*) left a large pool of susceptible individuals despite the high efficacy of the vaccine (*11*). In addition, abundances of the urban mosquito *Ae. aegypti* were heavily reduced at the start of the YFV epidemic because of extensive vector control efforts in response to the 2015–2016 Zika outbreaks (*7*, *12*). Together, these factors suggest that sylvatic mosquito vectors were carrying more transmissible strains of the virus and living in closer proximity to dense human populations with limited vaccine coverage (*6*). Against this backdrop, an extreme drought (*7*, *13*) may have drove the outbreak by increasing contact between infected mosquitoes and humans, consistent with models identifying low vaccine coverage, high population density, and climate factors as key drivers of YFV spillover risk (*14*).

Given the role of water in the mosquito life cycle, there is an intuitive reason to suspect excess precipitation to increase YFV incidence; however, drought could also influence disease dynamics by concentrating hosts and vectors in the same locations and times and/or by increasing mosquito feeding to counteract dehydration. The YFV epidemic peaked during the rainy seasons in 2017 and 2018; during the extreme drought (*7*, *13*), the modest rainfall during those periods may have intensified feeding and contact: Water-scarce conditions drive both mosquitoes and nonhuman primates to travel farther for water, with mosquitoes biting more to avoid desiccation. During such scarcity, highly habitat-adaptable and YFV-susceptible nonhuman primates, particularly howler monkeys and marmosets, are likely to concentrate in high densities in urban and peri-urban areas (*15*–*18*), where high infection rates were observed (*1*). Similarly, *Haemagogus* forest mosquitoes, capable of flying long distances in search of food and water (*19*), would similarly move toward urban areas and increase biting under dehydration stress (*20*). Multiple lines of evidence show that forest mosquitoes, primarily in the genus *Haemagogus*, were responsible for YFV transmission during this epidemic (*1*, *16*, *18*, *21*, *22*). This concentration of nonhuman primates and forest mosquitoes could therefore amplify transmission of YFV within their own populations and in closer proximity to humans (*7*). Drought-induced migration of nonhuman primates and forest mosquitoes into urban areas could therefore result in a major human outbreak. This hypothesis is consistent with other studies suggesting that changing environmental conditions and increased contact between nonhuman primates, forest mosquitoes, and humans propagated this unusual outbreak (*2*, *22*). Despite the biological plausibility of extreme drought triggering this YFV outbreak and other examples of drought concentrating pathogens, animals, and humans to amplify infectious disease transmission, drought has not previously been implicated in YFV outbreaks.

**Fig. 1.**
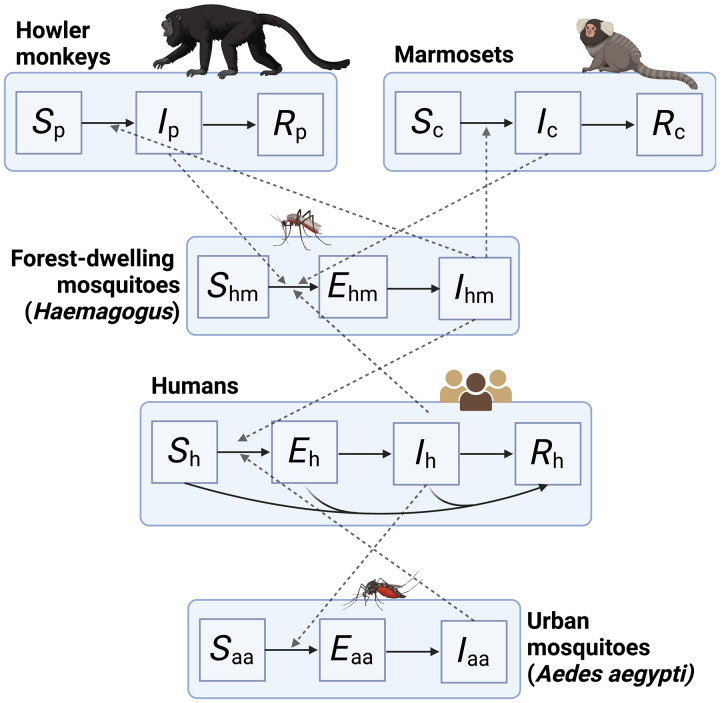
Compartmental model of YFV transmission in Brazil. Each blue Rectangle indicates a different species involved in disease transmission. Individuals within each species can move sequentially through several health states: susceptible (*S*), exposed (*E*), infectious (*I*), and removed (*R*). Nonhuman primates do not include an exposed state because it is unknown whether they have a latent period and how long it would last. Mosquitoes do not have a removed state because their lifespan is short relative to the study period. Solid arrows indicate the direction individuals move between compartments, and dashed arrows indicate the direction of transmission. The curved arrows in the human population represent vaccination. Created in BioRender. Caldwell, J. (2026), https://biorender.com/y0g3g38.

**Fig. 2.**
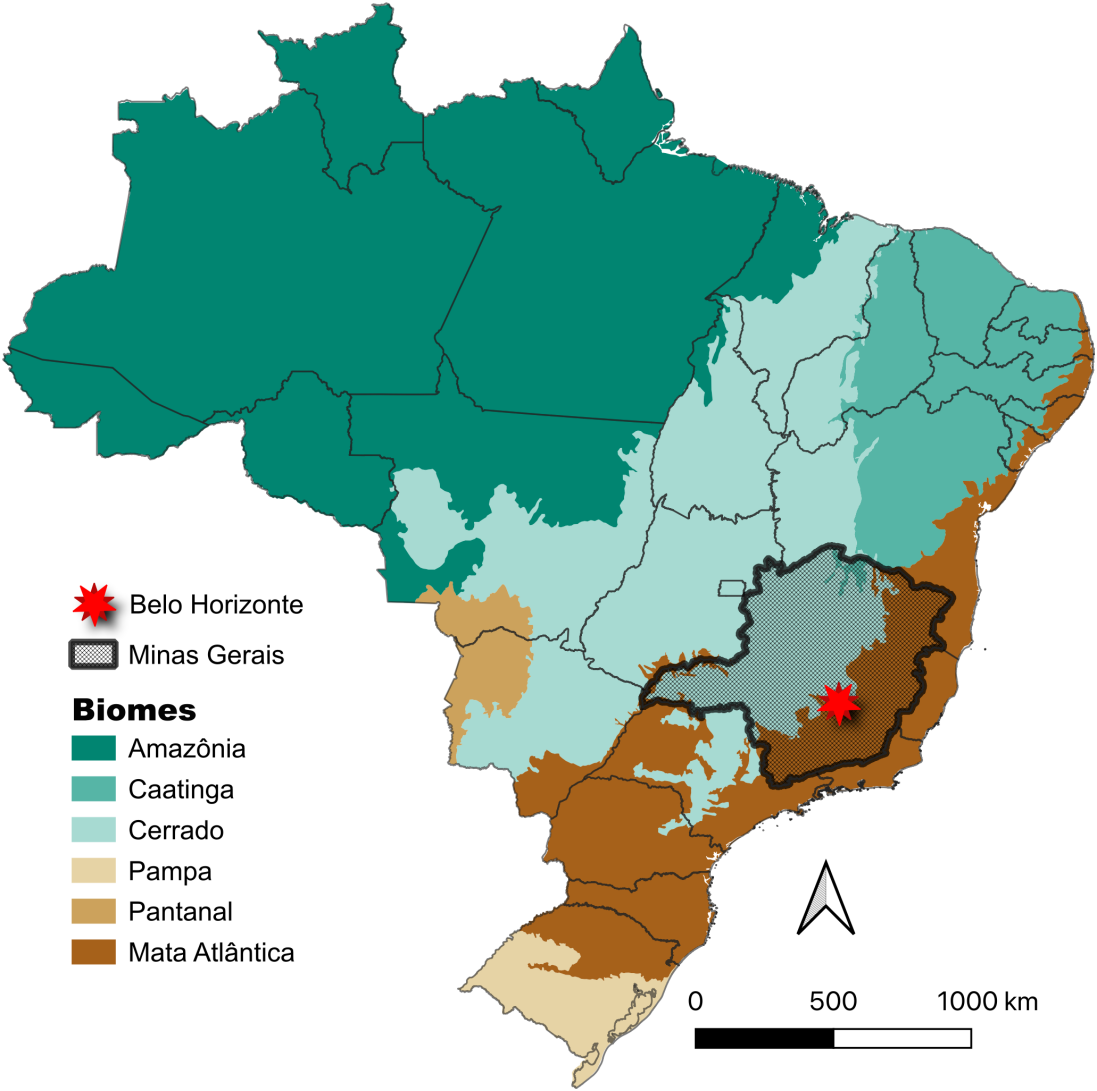
YFV outbreak of 2017–2018 occurring in Minas Gerais, Brazil. The 2017–2018 YFV outbreak in southeastern Brazil started and predominantly occurred in the state of Minas Gerais. Minas Gerais is divided between two predominant biomes, Cerrado and Mata Atlântica, with the capital city Belo Horizonte situated on the border of those biomes. Brazil is composed of six terrestrial biomes, which have unique plant and animal profiles created by their climate and geomorphological characteristics. Cerrado is a tropical savanna with grass, shrubs, and trees; large sections have been converted for soy and beef production. Mata Atlântica is a highly fragmented humid coastal tropical and subtropical forest. The other biomes of Brazil include the following: Amazônia, tropical rainforest; Caatinga, semiarid scrubland; Pampa, savanna with temperate grasslands and low shrubs; Pantanal, alluvial flood plain with sandy terrain. Brazil state boundaries data source: geoBoundaries version 6.0.0, CIESIN/William & Mary; licensed under CC BY 4.0 (https://creativecommons.org/licenses/by/4.0/). Brazil biome boundaries data source: Global Forest Watch, World Resources Institute, Brazil Biomes dataset, licensed under CC BY 4.0 (https://creativecommons.org/licenses/by/4.0/).

To evaluate the extent to which seasonally driven mosquito feeding and nonhuman primate incursion into the cities could have contributed to the 2017–2018 outbreak and assess intervention strategies, we simulated YFV transmission with a compartmental model parameterized on the basis of different hypotheses and compared those outputs with real-world evidence. We developed a compartmental model that captures key features of the Brazil YFV epidemic, including transmission from *Haemagogus* and *Ae. aegypti* mosquitoes to nonhuman primates (howler monkeys and marmosets) and humans (Fig. 1). Within this model structure, we asked how well the model reproduced disease dynamics in Minas Gerais (Fig. 2) during the epidemic with nested models accounting for seasonally driven mosquito feeding behavior, movement of forest nonhuman primates and mosquitoes, or both. Then, we assessed the extent to which different interventions could have reduced the second wave of transmission and whether those interventions would carry into the future.

## RESULTS

### Comparison of seasonal drivers

**Fig. 3.**
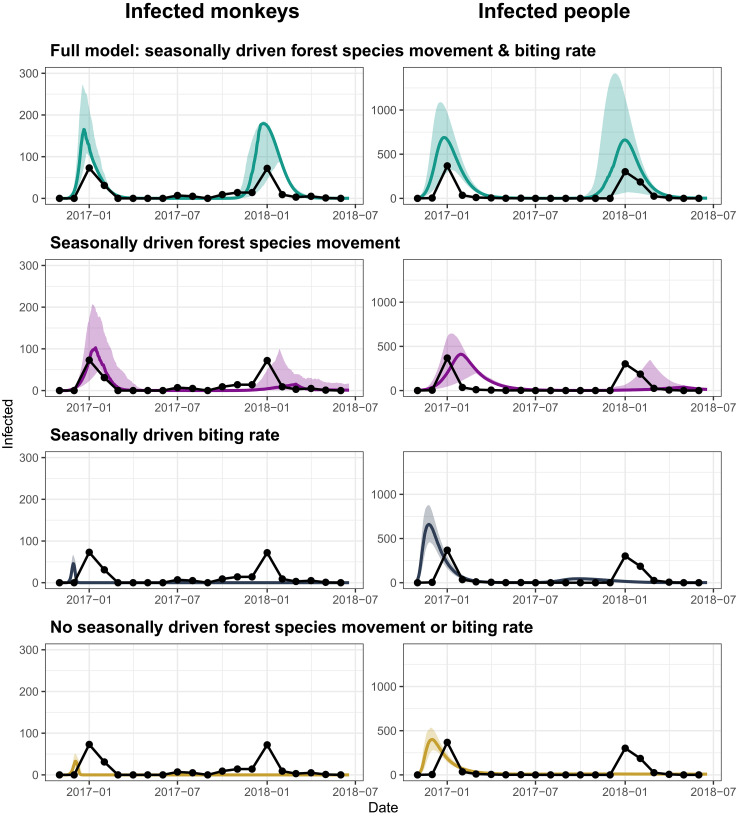
Seasonally driven forest species movement and mosquito biting contributed to driving the two-wave YFV epidemic in Brazil. Comparison of four nested models with observations for nonhuman primate cases (left) and human cases (right; lead time = 1 month). The full model (teal) included seasonally driven forest species movement and mosquito biting. The model with seasonally driven mosquito biting (purple) holds forest species movement constant, while the model with seasonally driven forest species movement (dark blue) holds seasonally driven mosquito biting constant; both models did not predict a second epidemic peak. The model that holds both seasonally driven forest species movement and mosquito biting constant (yellow) predicted an early first wave and no second wave of transmission. We show observations overlaid for comparison (black dots connected by lines).

The model that incorporated both seasonally driven mosquito biting behavior and movement of forest species (hereafter referred to as the full model) reproduced the observed 2-year epidemic dynamics in both nonhuman primates and humans better than the simplified, nested model variants (Fig. 3 and Table 1). We evaluated model performance using both correlation with observed incidence through time and normalized root mean square error (NRMSE), which quantifies the average prediction error relative to the scale of the observed data and allows comparisons across outputs with different magnitudes. The full model captured the epidemic seasonality well, and sensitivity analyses support the robustness of these conclusions (fig S1 and table S2). If either or both seasonal components were excluded from the model, the predictions poorly predicted nonhuman primate epidemic dynamics and incorrectly reproduced the timing of the first epidemic wave (either too early or late) while failing to predict a second wave of transmission in humans. The nested models that incorporated seasonally driven mosquito biting but restricted the movement of forest species predicted no transmission in nonhuman primates and an early first wave of human transmission. Furthermore, the model that included only seasonally driven mosquito biting overpredicted human incidence in the first wave; it did however also predict a small increase in cases that did not materialize into an outbreak about 4 months before the second wave of transmission. In contrast, the model that incorporated seasonally driven forest species movement but restricted mosquito biting predicted a late first wave of human transmission; this model did show some uncertainty around the second wave of transmission at the correct time. These results indicate that both increased mosquito biting during the rainy season and increased movement of forest species into the cities, partially driven by the ongoing drought conditions, were needed for the two-peak epidemic to develop. A global sensitivity analysis, which allowed all parameters (both seasonal and nonseasonal) to vary simultaneously, indicated that seasonally driven mosquito births and deaths, which vary with climatic conditions, were highly influential as well. Together, the model comparison and sensitivity analyses provide complementary lines of evidence that seasonal environmental factors, especially those influencing mosquito survival, biting, and host/vector movement, were the dominant drivers of YFV outbreak timing and magnitude during this epidemic.

**Table 1.** Correlation (*P* value) and NRMSE between model predictions and observations across nested seasonally driven models. Modeled human cases were adjusted by a 1-month lead time. NHP, nonhuman primates. Note that the “No seasonality” model still includes a seasonal mosquito carrying capacity.

Model	Human cases	NHP cases	Avg. correlation and NRMSE
Full model	0.90 (*P* < 0.001); 4	0.90 (*P* < 0.001); 3	0.90; 3.5
Seasonally driven forest species movement only	0.22 (*P* > 0.05); 3	0.68 (*P* < 0.05); 2	0.45; 2.5
Seasonally driven mosquito biting only	0.15 (*P* > 0.05); 4	−0.10 (*P* > 0.05); 2	0.03; 3
No seasonality	0.23 (*P* > 0.05); 3	−0.10 (*P* > 0.05); 2	0.07; 2.5

### Optimizing interventions

**Fig. 4.**
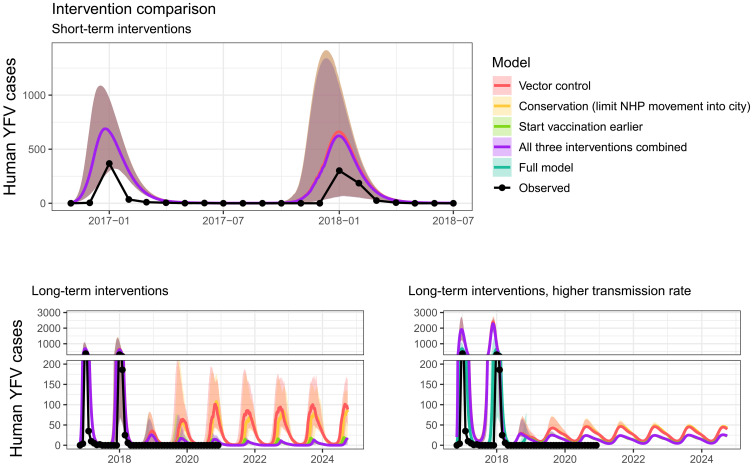
Deploying multiple control strategies simultaneously allow for sustained YFV control. Effects of interventions in the short term (top) and long term (bottom). We simulated four intervention strategies: vector control (i.e., reducing mosquito populations in the rainy season; pink), conservation initiatives (i.e., reducing forest species movement into the cities; orange), early and continued vaccine distribution (green), and a combination of all three (purple). For comparison, we show observations (black points connected by lines) and the full model (teal). NHP, nonhuman primates.

Using multiple interventions simultaneously had the highest likelihood of minimizing YFV transmission in the long term but would not have been enough to prevent the second epidemic wave (Fig. 4). The intervention that reflected spraying insecticides every rainy season, the key vector control tactic used in the region, led to a reduction in cases in the shorter term but allowed for increasingly large annual outbreaks resulting from reaccumulation of mosquito populations. The intervention that reflected conservation initiatives aimed at reducing the movement of forest species to cities had a similar effect. The third intervention initiated the vaccination campaign at the onset of the epidemic (whereas, in reality, the campaign was delayed until after the first peak) and then continued YFV vaccination as part of routine childhood vaccinations in the region; this intervention had the strongest effect overall, with increasing effectiveness through time, contrasting the patterns of the first two interventions. The simulation that included all three interventions showed substantial and sustained control of YFV, which was largely driven by vaccination efforts. The other interventions still remain valuable though; if the virus evolved to evade the vaccine, or vaccine uptake or distribution were heterogeneous (as is often the case), the other interventions would provide a substantial buffer. In the hypothetical situation where the virus evolved to be 50% more transmissible, which would be possible in the future, we would expect much larger initial outbreaks and the need for stronger integrated control. Unexpectedly, none of the interventions were strong enough to curb transmission in the short term.

### Extreme drought and global change

**Fig. 5.**
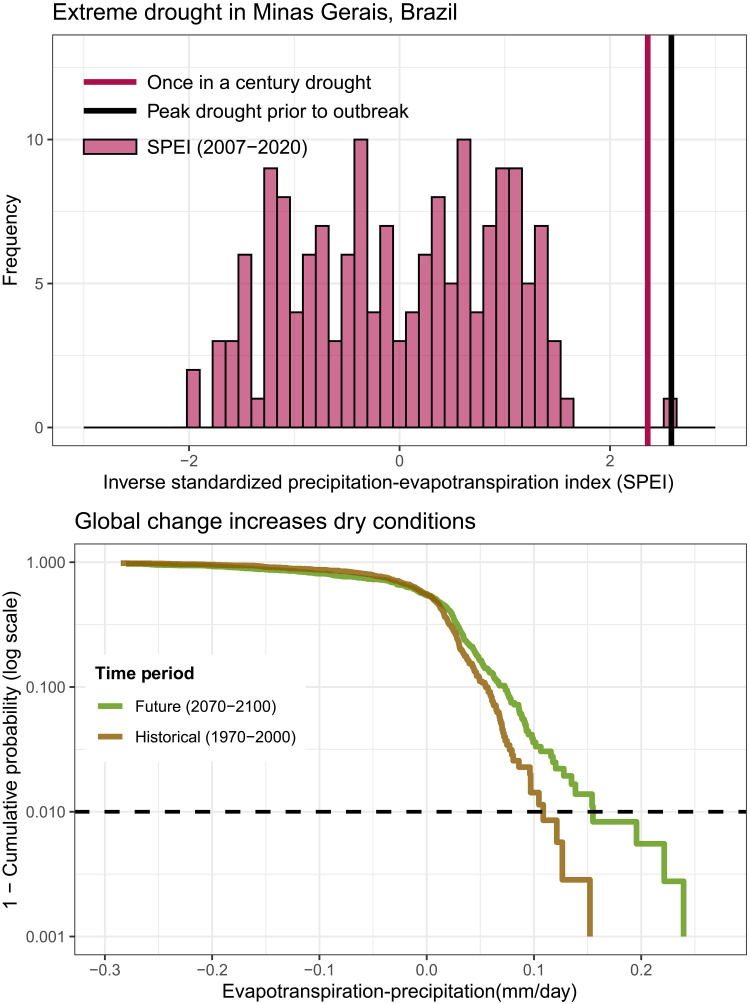
Conditions preceding the YFV outbreak were more extreme than a once in a century drought and global change will give rise to more extreme dry conditions in the future. Histogram of the inverse SPEI for Minas Gerais, Brazil (top). We show the value for a once in a century drought (pink vertical line) and the value for SPEI preceding the YFV outbreak (black vertical line). A cumulative distribution function (CDF) plot (bottom) shows the survival probability (1 − CDF) of the difference between evapotranspiration and precipitation (mm/day) for two time periods: historical (1970 to 2000) and future (2070 to 2100). We plotted the *y* axis in log space to highlight extreme events, with a dashed horizontal line indicating a 1% exceedance threshold. The plot illustrates an increased likelihood of extreme dry conditions in the future compared to the historical period. For top and bottom plots, negative numbers indicate wetter conditions, and positive numbers indicate drier conditions.

The drought before the 2017–2018 YFV outbreak in Minas Gerais, Brazil, was 9.5% more extreme than a once in a century drought (i.e., 100-year return period), and dry conditions are projected to increase in the future (Fig. 5). On the basis of the widely used drought metric, standardized precipitation-evapotranspiration index (SPEI), we show that extreme dry conditions were present 4 months before the YFV outbreak. This timeline aligns with the expected biophysical process that links climate to disease transmission, whereby dry conditions promote migration of nonhuman primates and forest mosquitoes toward city centers in search of food and water in the months preceding the outbreak. On the basis of climate models, future conditions in this region are 47% drier than historical conditions at the 1% exceedance threshold, indicating a substantial increase in the severity of extreme drought events (on the basis of projections with intermediate greenhouse gas emissions: SSP245). The IPCC-AR6 supports the notion that drought should increase in Brazil [figure 12.4 in (*23*)].

## DISCUSSION

YFV outbreaks are notoriously difficult to predict, and retrospective analyses can provide some insight into why outbreaks begin and why they end. The 2017–2018 outbreak in southeastern Brazil was particularly unusual because there had not been transmission in the region in nearly a century. Prior studies hypothesized that slow changes (e.g., buildup of a highly susceptible population and land use change) provided the background conditions for an outbreak to emerge (*14*). While these factors may explain why the region was vulnerable, we focused on how the outbreak unfolded, testing the hypothesis that short-term, seasonal climate conditions shaped the two-peak epidemic trajectory. By comparing models representing different hypotheses about seasonal drivers with real-world observations, we show that the combined effects of seasonally driven mosquito feeding behavior and seasonally driven forest species incursion into the city explain the outbreak timing and incidence. While factors such as low population immunity and land use change likely shaped the broader susceptibility landscape, our results demonstrate that seasonal ecological processes were key to explaining the timing and structure of transmission during the outbreak. Even in the absence of drought conditions, it is possible that once seeded, an outbreak would have taken off because of high susceptibility in the population. While seasonal climate appears to explain how the outbreak developed, it is equally important to understand why the outbreak did not continue to cause annual epidemics thereafter. The intervention simulations show that a combination of activities likely contained the outbreak but could not have avoided the second peak in transmission. In reality, the three techniques described (vector control, conservation, and vaccination) were used to different extents and largely align with real-life observations, where YFV cases were not reported after 2018. The simulations also suggest that there could be very low levels of continued transmission. Given the high rate of asymptomatic transmission (*24*), it is plausible that some cases go undetected every year.

The Brazilian epidemic was also unusual because it spread in urban areas but was predominantly spread by a nonurban mosquito species, amplified by nonhuman primate transmission. The outbreak was driven by *Haemagogus* rather than *Ae. aegypti* mosquitoes. The expectation was that given sufficient cases, there would be reestablishment of an urban transmission cycle perpetuated by *Ae. aegypti*-human transmission as *Ae. aegypti* remains a competent YFV vector in laboratory studies (*25*, *26*). Using a two-vector model, we show that although *Ae. aegypti* are competent YFV vectors, they did not meaningfully contribute to transmission during this epidemic, likely due to their strong anthropophilic feeding behavior. This finding aligns with entomologic surveys conducted during the outbreak, which failed to detect YFV-infected *Ae. aegypti* despite extensive sampling (*27*–*30*). If *Ae. aegypti* were to become a significant YFV vector in the future, drought conditions could further amplify transmission by increasing the use of water storage containers, which serve as ideal breeding sites for this species. However, during this epidemic, water storage practices were unlikely to drive transmission, as *Haemagogus* mosquitoes, identified as the primary vectors, prefer natural oviposition sites such as tree holes located several meters above the ground (*31*). Consistent with this interpretation, removing *Ae. aegypti* from the model did not substantially alter transmission dynamics (table S3). This outbreak was driven by *Haemagogus* flexibly feeding on alternate hosts, which has been reported previously (*32*–*34*), and was likely bolstered by feeding on nonhuman primates in the city, which have higher and longer viremia profiles than people (*1*, *2*), indicating that even low abundances of nonhuman primates can affect transmission. The contribution of nonhuman primates in urban transmission may also be the reason why vaccination alone did not appear adequate to reduce transmission in the short term. Our expectation was that to limit outbreaks (i.e., *R*_0_ < 1, where *R*_0_ is the basic reproduction number), vaccination coverage needed to be 76 to 79% [coverage required to limit outbreaks = 1 − 1/*R*_0_, where median *R*_0_ = 4.21 and mean *R*_0_ = 4.81 across multiple studies (*35*)]. In this study, we allowed vaccination coverage to reach 90%, yet the simulations showed that vaccination alone could not have prevented the second wave of transmission, suggesting that we should consider the susceptible population as a combination of humans and nonhuman primates. A related important consideration is that continued viral evolution will increase the critical population size necessary for disease control. Thus, a suite of integrated control measures will become increasingly important.

Land use change was likely the proximate cause of nonhuman primate incursion into the city, and ecological countermeasures may be key to reducing future disease emergence risk. While the model does not explicitly incorporate spatial heterogeneity across forest, peri-urban, and urban settings because of limited habitat-specific data availability, which limits the ability to resolve edge effects and spatial transmission dynamics, deforestation and other types of habitat alteration are known to increase spillover risk of disease from the forest to the city by affecting nonhuman primate susceptibility and spatial behavior (*36*, *37*). Reduced habitat quality and area often limit food and water availability, forcing nonhuman primates to invest extra physiological resources for basic survival, leading to reduced immune function. Immune dysregulation can both increase susceptibility to infection and increase pathogen loads and shedding time (*36*). In addition, animals may need to expand their search area to find food (*36*, *38*–*40*). These behavioral changes then increase the likelihood and length of infected nonhuman primate-*Haemagogus* contact within cities where *Haemagogus* are also biting people. Ecological countermeasures, or conservation initiatives, are therefore an effective strategy for primary protection against spillover events; such measures include protecting and restoring wildlife habitat, creating buffer zones, and strategically safeguarding critical habitat for animal feeding, resting, and social aggregation, ultimately strengthening barriers that separate (infected) wildlife and vectors from people (*36*).

Another pillar of epidemic prevention is vaccination. The YFV vaccine is highly efficacious, well studied, and largely available to high-risk populations (*11*). Given that southeastern Brazil had not experienced a YFV outbreak in nearly a century, most of the region was not considered high risk and was undervaccinated at the start of the epidemic (*6*). Perhaps more problematic than undervaccination though was that in parts of Minas Gerais where YFV vaccination programs were in place before the 2017–2018 outbreak, coverage was incomplete and levels of neutralizing antibodies were undetectable across a large proportion of the population (*9*, *10*), including in individuals with a documented history of YFV vaccination (*9*). Undetectable levels of neutralizing antibodies indicate waning immunity, which is unexpected given that the World Health Organization has deemed that a single dose of the YFV vaccine confers lifelong immunity but plausible given some other studies (*41*). This is an important finding as vaccines that do not confer lifelong immunity require different vaccine schedules than those that do and have different implications for long-term disease dynamics. Additional issues that should be considered in epidemic prevention planning are potential bottlenecks such as vaccine shortages, cold transport delays, or distribution problems.

The central premise of this study was to investigate the role that drought may have played in driving outbreak dynamics, building on a growing body of evidence linking precipitation and disease, to ultimately understand how climate and climate change affect infectious disease dynamics. Among the different climate change–related hazards that can affect climate-sensitive infectious diseases, drought is a relatively difficult one to examine. Unlike heatwaves or floods, which are usually distinct events, drought conditions build slowly and can last months to years or more. As a result, robustly linking drought to an epidemic is difficult because of the differing timescales. Despite this challenge, drought has been implicated in other water-borne, vector-borne, and zoonotic disease outbreaks by concentrating humans, animals, and pathogens around limited water sources (*42*–*45*). In this study, we provide support for two mechanisms for this relationship: (i) Drought can cause nonhuman primate hosts and competent mosquito vectors to move toward human inhabited spaces, and (ii) mosquito vectors bite more frequently to avoid desiccation, leading to more opportunities for transmission. Although the model supports a role for seasonally driven movement in shaping epidemic dynamics, we did not mechanistically couple this process to climatic variables such as the SPEI because of limitations in spatial and temporal resolution of data and the likely nonlinear and lagged behavioral responses to drought. A more explicit drought-movement linkage would require habitat-specific estimates of primate and mosquito movement, vector-host contact rates, and fine-scale environmental data, which were unavailable for this outbreak but would greatly strengthen future inference. Climate change allows droughts to set in quicker and become more intense, so we would expect drought-disease dynamics to become more prominent, yet there are many challenges in developing reliable drought assessments under climate change, making our ability to anticipate such events nontrivial (*46*). On the other hand, the slow buildup of drought relative to other events with short lead times like cyclones and floods provides an opportunity for anticipatory action from health systems as soon as drought conditions start to build. This knowledge could therefore be used to develop early warning systems to better support decision-makers and health system planning, both for YFV and potentially other arboviruses. Adopting a One Health approach that combines land and water conservation with early outbreak identification and vaccination could help prevent or control outbreaks. Such systems would be useful beyond Brazil, as other countries like Bolivia, Colombia, Guyana, and Peru face similar deforestation, primate displacement, vaccination, and climate change issues and have recently seen an uptick in YFV reports (WHO Disease Outbreak News).

## METHODS

### Model and parameters

We developed a compartmental model to simulate transmission dynamics for YFV in Brazil, accounting for key features of the dynamical system. It includes both *Haemagogus* and *Ae. aegypti* mosquitoes as vectors and howler monkeys (genus *Aloutta*), marmosets (genus *Calithrix*), and humans as hosts (Fig. 1). Within each species, individuals move between some combination of susceptible, exposed, infectious, and removed (i.e., recovered/dead) states (Fig. 1). The model represents transmission in the city, and population estimates reflect our understanding of population dynamics around the time of the epidemic: *Ae. aegypti* abundances were approximately two to three times lower than in the previous decade because of extensive vector control efforts in response to the Zika pandemic [on the basis of a Rapid Index Survey for *Ae. aegypti* by the Prefeitura Do Rio de Janeiro (accessed 13 April 2021)]. We estimated *Haemagogus* mosquito abundances as a fraction (55 to 85%) of the *Ae. aegypti* population because *Haemagogus* mosquitoes typically live in the forest and bite nonhuman primates in the canopy, only descending and moving to bite people when the forest environment is disturbed or canopy hosts are scarce (*33*, *34*). In addition, we allowed for seasonally varying mosquito carrying capacity, birth rates, and biting rates on the basis of the rainy season. Similarly, we allowed seasonal immigration of howler monkeys and marmosets from the forest to the city as they are highly adapted to fragmented peri-urban habitats (*15*, *17*, *18*, *21*), and many more carcasses were found in and around the city than a typical year (*7*). While we hypothesize that drought-induced ecological stress drove forest mosquitoes and nonhuman primates toward urban areas, the available climate and behavioral data were insufficient to mechanistically link climatic indices (e.g., SPEI) directly to movement dynamics in the model. Instead, we represent such seasonal ecological forcing as a proxy for temporally varying pressure on host and vector movement.

The model tracks the number of individuals in the following epidemiological compartments: susceptible (*S*), exposed (*E*, where applicable), infectious (*I*), and recovered/dead (*R*, where applicable). The compartment subscripts refer to the population group: howler monkeys (p), marmosets (c), humans (h), *Haemagogus* mosquitoes (hg), and *Ae. aegypti* mosquitoes (aa). For each population group *x*, the total population size is given by the sum of its compartments

$$N_{x}=S_{x}+E_{x}+I_{x}+R_{x}$$ (1)

We describe the set of ordinary differential equations below with associated parameter definitions and values in Table 2.

**Table 2.** Model parameter symbols, definitions, values, and sources. Numbers in brackets indicate low and high estimates used for seasonal forcing and/or in the sensitivity analysis. Note that λ_x_ represents derived force of infection where x indicates the population of interest. Pi (π) is a mathematical constant that equals approximately 3.14.

Parameter	Definition	Value	Source
α	Mosquito biting rate (*Haemagogus* and *Aedes*)	0.70 [0.30, 1.00]	(*49*–*51*)
pMI_1_	Probability of *Haemagogus* infection from infectious monkey	0.20 [1.00]	(*52*)
pMI_2_	Probability of *Haemagogus* infection from infectious human	0.25 [0.50]	(*16*)
pMI_3_	Probability of *Aedes* infection from infectious human	0.25 [0.50]	(*51*)
pMI_4_	Probability of *Haemagogus* infection from infectious marmoset	0.125 [0.25]	(*16*)
*b*	Probability mosquito becomes infectious after infection	0.50	(*51*)
PDR_hg_	YFV development rate in *Haemagogus*	8.3 × 10^-2^	(*53*, *54*)
PDR_aa_	YFV development rate in *Aedes*	7.7 × 10^-2^	(*55*–*57*)
μ_hg_	*Haemagogus* birth/mortality rate	0.05 [3.70 × 10^-2^, 1.43 × 10^-1^]	(*58*–*60*)
μ_aa_	*Aedes* birth/mortality rate	0.05 [2.86 × 10^-2^, 0.10]	(*51*, *61*)
μ_p_	Primate birth/mortality rate	1.6 × 10^-4^	(*62*)
μ_c_	Marmoset birth/mortality rate	4.6 × 10^-4^	(*63*)
μ_h_	Human birth/mortality rate	3.7 × 10^-5^	(World Bank)
μ_v1_	Mortality rate of primates with YFV	0.50 [0.20, 0.80]	(*16*)
μ_v2_	Mortality rate of marmosets with YFV	0.01	(*16*)
μ_v3_	Mortality rate of humans with YFV	0.02	(*56*)
γ_p_	Primate recovery rate (1/infectious period)	1.7 × 10^-1^	(*52*, *64*, *65*)
γ_c_	Marmoset recovery rate (1/infectious period)	0.25	(*66*)
γ_h_	Human recovery rate (1/infectious period)	2.9 × 10^-1^	(*51*, *52*)
δ_h_	Human incubation rate (1/incubation period)	0.23	(*56*, *57*, *67*)
*K*	Mosquito carrying capacity	[4.0 × 10^5^, 7.0 × 10^6^]	(This study)
*V*	Vaccination rate (per day)	[0.00, 6.7 × 10^-4^]	(*68*, *69*)
*m*_1_	Movement of howlers from forest to city (#/month)	100 [5, 200]	(This study)
*m*_2_	Movement of marmosets from forest to city (#/month)	75 [10, 150]	(This study)
*m*_3_	Movement of *Haemagogus* mosquitoes from forest to city (#/month)	10 [1, 100]	(This study)

#### Howler monkeys

$$\frac{{\mathrm{dS}}_{p}}{\mathrm{dt}}=-\alpha \left[t\right]b\frac{I_{\text{hg}}}{N_{p}}S_{p}+\mu_{p}\left(N_{p}-S_{p}\right)+m_{1}\left[t\right]$$ (2)

$$\frac{{\mathrm{dI}}_{p}}{\mathrm{dt}}=\alpha \left[t\right]b\frac{I_{\text{hg}}}{N_{p}}S_{p}-\gamma_{p}I_{p}-\mu_{p}I_{p}-\mu_{v1}I_{p}$$ (3)

$$\frac{{\mathrm{dR}}_{p}}{\mathrm{dt}}=\gamma_{p}I_{p}-\mu_{p}R_{p}$$ (4)

#### Marmosets

$$\frac{{\mathrm{dS}}_{c}}{\mathrm{dt}}=-\alpha \left[t\right]b\frac{I_{\text{hg}}}{N_{c}}S_{c}+\mu_{c}\left(N_{c}-S_{c}\right)+m_{2}\left[t\right]$$ (5)

$$\frac{{\mathrm{dI}}_{c}}{\mathrm{dt}}=\alpha \left[t\right]b\frac{I_{\text{hg}}}{N_{c}}S_{c}-\gamma_{c}I_{c}-\mu_{c}I_{c}-\mu_{v2}I_{c}$$ (6)

$$\frac{{\mathrm{dR}}_{c}}{\mathrm{dt}}=\gamma_{c}I_{c}-\mu_{c}R_{c}$$ (7)

#### Humans

$$\frac{{\mathrm{dS}}_{h}}{\mathrm{dt}}=-\lambda_{h}S_{h}+\mu_{h}\left(N_{h}-S_{h}\right)-V\left[t\right]S_{h}$$ (8)

$$\frac{{\mathrm{dE}}_{h}}{\mathrm{dt}}=\lambda_{h}S_{h}-\delta_{h}E_{h}-\mu_{h}E_{h}-V\left[t\right]E_{h}$$ (9)

$$\frac{{\mathrm{dI}}_{h}}{\mathrm{dt}}=\delta_{h}E_{h}-\gamma_{h}I_{h}-\mu_{h}I_{h}-\mu_{v3}I_{h}-V\left[t\right]I_{h}$$ (10)

$$\frac{{\mathrm{dR}}_{h}}{\mathrm{dt}}=\gamma_{h}I_{h}-\mu_{h}R_{h}+V\left[t\right]\left(S_{h}+E_{h}+I_{h}\right)$$ (11)

where

$$\lambda_{h}\left[t\right]=\alpha \left(b\frac{I_{\text{hg}}}{N_{h}}+\frac{I_{\text{aa}}}{N_{h}}\right)$$

#### Haemagogus *mosquitoes*

$$\frac{{\mathrm{dS}}_{\text{hg}}}{\mathrm{dt}}=-\lambda_{\text{hg}}S_{\text{hg}}+\mu_{\text{hg}}\left[t\right]\left(N_{\text{hg}}-S_{\text{hg}}\right)\left(1-\frac{N_{\text{hg}}}{K\left[t\right]}\right)+m_{3}\left[t\right]$$ (12)

$$\frac{{\mathrm{dE}}_{\text{hg}}}{\mathrm{dt}}=\lambda_{\text{hg}}S_{\text{hg}}-\left({\text{PDR}}_{\text{hg}}+\mu_{\text{hg}}\right)E_{\text{hg}}$$ (13)

$$\frac{{\mathrm{dI}}_{\text{hg}}}{\mathrm{dt}}={\text{PDR}}_{\text{hg}}E_{\text{hg}}-\mu_{\text{hg}}I_{\text{hg}}$$ (14)

where

$$\lambda_{\text{hg}}\left[t\right]=\alpha \left[t\right]\left({\text{pMI}}_{1}\frac{I_{p}}{N_{p}}+{\text{pMI}}_{2}\frac{I_{h}}{N_{h}}+{\text{pMI}}_{4}\frac{I_{c}}{N_{c}}\right)$$

#### Ae. aegypti *mosquitoes*

$$\frac{{\mathrm{dS}}_{\text{aa}}}{\mathrm{dt}}=-\lambda_{\text{aa}}S_{\text{aa}}+\mu_{\text{aa}}\left[t\right]\left(N_{\text{aa}}-S_{\text{aa}}\right)\left(1-\frac{N_{\text{aa}}}{K\left[t\right]}\right)$$ (15)

$$\frac{{\mathrm{dE}}_{\text{aa}}}{\mathrm{dt}}=\lambda_{\text{aa}}S_{\text{aa}}-\left({\text{PDR}}_{\text{aa}}+\mu_{\text{aa}}\right)E_{\text{aa}}$$ (16)

$$\frac{{\mathrm{dI}}_{\text{aa}}}{\mathrm{dt}}={\text{PDR}}_{\text{aa}}E_{\text{aa}}-\mu_{\text{aa}}I_{\text{aa}}$$ (17)

#### Carrying capacity

$$K\left[t\right]=\frac{K_{\text{max}}+K_{\text{min}}}{2}+\frac{K_{\text{max}}-K_{\text{min}}}{2}\text{cos}\left(\frac{4t\pi }{\text{days}}\right)$$ (18)

### Simulations

To assess the role of rainfall seasonality in driving the YFV epidemic, we simulated disease transmission with four models with different seasonal drivers. The full model included seasonally driven carrying capacity, mosquito biting rates, and movement (i.e., immigration) of forest species to the city. Carrying capacity is commonly treated as seasonal [e.g., (*47*)], whereas the pervasiveness and importance of seasonally driven biting rates and movement are less well understood. Thus, the three other models tested the importance of these factors in nested versions of the full model: Model two included seasonally driven movement of nonhuman primates and *Haemagogus* mosquitoes but held mosquito biting rate constant, model three included seasonally driven mosquito biting but limited movement of forest species, and model four excluded both seasonally driven movement of forest species and mosquito biting.

Given that some parameter values are less certain than others, we assessed the sensitivity of the model outcomes to the most uncertain parameter values. In the sensitivity analysis, we evaluated the impact of increasing and decreasing three highly uncertain parameters, resulting in five model specifications. The five models used the full model but replaced the following parameter values: models 1 and 2, low/high YFV-induced monkey mortality rates; model 3, high probability of mosquito infection with YFV (simultaneously increasing the four pMI values); models 4 and 5, low/high movement rate of forest species to the city.

To assess the extent to which different interventions could have prevented the second wave of the YFV epidemic and beyond, we generated four model specifications representing (i) vector control (i.e., reducing mosquito populations by 50% via insecticide spraying in rainy seasons), (ii) conservation measures to reduce the movement of forest species into urban habitats, (iii) earlier deployment of vaccines (3 months earlier than actual deployment, corresponding with the initial outbreak and continuing with early childhood vaccination campaigns), and (iv) a combination of all three interventions. We ran the intervention simulations for 8 years to assess whether interventions were effective in the short and long term. We additionally ran all four intervention simulations assuming that *R*_0_ values were doubled to understand the effectiveness of the interventions if the virus were more transmissible.

For all 17 model specifications [four assessing the role of seasonality, five assessing parameter uncertainty, and eight assessing interventions (four interventions * two *R*_0_ levels)], we ran each simulation 500 times to account for initial condition uncertainty (i.e., compartment values at the start of the simulation period). We show the distributions for these starting values in table S1.

### Global sensitivity analysis

To evaluate the relative importance of model parameters and their interactions, we conducted a global sensitivity analysis using Latin hypercube sampling combined with partial rank correlation coefficients (PRCCs). We generated 1000 unique parameter sets using Latin hypercube sampling across biologically plausible ranges (±10%) for 26 model parameters (see table S3), including both seasonal parameters (e.g., mosquito mortality μ_hg_, biting rate α, carrying capacity *K*, and movement rates μ_1−3_) and nonseasonal parameters (e.g., transmission probability *b*, recovery rates γ, mortality rates μ, and incubation periods δ). We simulated the full model under each parameter set and computed four summary metrics for correlation and NRMSE to assess model fit for both human and primate cases. We calculated PRCCs between each parameter and each model output to quantify monotonic relationships while controlling for other parameters. PRCC values range from −1 (strong inverse correlation) to +1 (strong positive correlation), and *P* values indicate statistical significance. Parameters with PRCC values ≥ 0.4 and *P* < 0.05 are considered highly influential.

### Data

We obtained monthly confirmed human and nonhuman primate cases in Minas Gerais from the Brazilian national health surveillance system, Sistema de Informação de Agravos de Notificação. Human cases were confirmed as acute YFV on the basis of serology or polymerase chain reaction. Nonhuman primates were tested for YFV by the Division of Zoonoses and Vector-borne Diseases. We used pooled cases of five genera of nonhuman primates, predominately from the genus *Aloutta* (i.e., howler monkeys) but also composed of *Calithrix* (i.e., marmosets), *Cebus*, *Saimiri*, and *Sapajus*. In the main analysis, we compared simulations with data for the outbreak (November 2016 to April 2017; *n* = 20 months of case counts for both humans and nonhuman primates). For visualization purposes, we show monthly aggregated human cases through 2020 in the intervention plots.

To contextualize the drought preceding the outbreak within historical and future climate conditions, we assessed two metrics of precipitation. We used the SPEI based on weather station data to assess observed conditions between 2007 and 2020 in Minas Gerais, Brazil, from (*7*). The index measures water balance, with negative numbers indicating a water deficit. To assess how dry conditions may change in the future in this region, we visualized distributions of historical and future mean monthly evapotranspiration minus precipitation from six models participating in the CMIP6 (Coupled Model Intercomparison Project Phase 6) (*48*): GFDL-CM4, GFDL-ESM4, CESM2-WACCM, CESM2, MPI-ESM1-2-HR, and MPI-ESM1-2-LR. For future projections, we used the SSP2-4.5 scenario, which represents a moderate pathway of greenhouse gas emissions. We estimated return periods by fitting a normal distribution to the precipitation data and using its parameters (mean and standard deviation) to calculate the value corresponding to an exceedance probability of 1/return period. This approach estimates the threshold value that corresponds to a specified frequency of exceedance. To compare the relative magnitude of two exceedance values (e.g., historical versus future 100-year return period), we calculated the ratio of the larger exceedance value to the smaller one. This ratio quantifies how many times the larger value exceeds the smaller one, providing a sense of their relative difference in terms of magnitude or return period.

### Validation

We evaluated how well each model specification (with the exception of intervention scenarios) generated YFV transmission dynamics by correlating time series of model simulations with observational data. We calculated correlation between monthly predictions and observations using the cross-correlation function in base R (statistical software). For the nonhuman primate comparison, we used no time lag, whereas for the human cases, we used a 1-month lag (incorporated as a 30-day lead time of modeled cases). We used a lag for human cases because the observational data were aggregated to monthly case counts, but in reality, cases could have occurred up to 30 days apart. Anecdotally, the infected nonhuman primate carcasses were found before the peak in human cases, suggesting a lag of a few weeks, despite the data showing peaks in the two populations in the same month each year. For each model specification, we calculated the correlation between observations (i.e., reported cases/deaths) and median model predictions for humans and nonhuman primates (howler monkeys only), adjusted on the basis of expected reporting rates [0.7 for nonhuman primates because mortality rates are high (*9*) and 0.45 for humans based on (*24*)]. In addition to correlation, we calculated the NRMSE to quantify the average magnitude of prediction error relative to the scale of observed values. NRMSE provides a complementary measure of model fit that captures absolute differences in magnitude and timing, rather than just shared shape or trend. A value of zero indicates perfect predictions. We determined the best-fit model as the one that best characterized both human and nonhuman primate infection dynamics (i.e., highest average correlation across the two categories) while also considering NRMSE as a supporting diagnostic.

## Supplementary Materials

### This PDF file includes:

Sensitivity analyses Analysis removing *Ae. aegypti* Figs. S1 to S3 Tables S1 to S3

## References

[1] N. I. O. Silva, L. Sacchetto, I. M. de Rezende, G. de Souza Trindade, A. D. LaBeaud, B. de Thoisy, B. P. Drumond, Recent sylvatic yellow fever virus transmission in Brazil: The news from an old disease. Virol. J. 17, 9 (2020). 31973727 10.1186/s12985-019-1277-7 PMC6979359

[2] L. Sacchetto, N. I. O. Silva, I. M. de Rezende, M. S. Arruda, T. A. Costa, É. M. de Mello, G. F. G. Oliveira, P. A. Alves, V. E. de Mendonça, R. G. A. V. Stumpp, A. I. A. Prado, A. P. Paglia, F. A. Perini, M. Lacerda Nogueira, E. G. Kroon, B. de Thoisy, G. de Souza Trindade, B. P. Drumond, Neighbor danger: Yellow fever virus epizootics in urban and urban-rural transition areas of Minas Gerais state, during 2017-2018 yellow fever outbreaks in Brazil. PLoS Negl. Trop. Dis. 14, e0008658 (2020). 33017419 10.1371/journal.pntd.0008658 PMC7535057

[3] J.-P. Chippaux, A. Chippaux, Yellow fever in Africa and the Americas: A historical and epidemiological perspective. J. Venom Anim. Toxins Incl. Trop. Dis. 24, 20 (2018). 30158957 10.1186/s40409-018-0162-y PMC6109282

[4] P. F. C. Vasconcelos, “Yellow fever” in *Arthropod Borne Diseases*, C. B. Marcondes, Ed. (Springer International Publishing, Cham, 2017), pp. 101–113; 10.1007/978-3-319-13884-8_8.

[5] S. C. Hill, S. Dellicour, I. M. Claro, P. C. Sequeira, T. Adelino, J. Thézé, C.-H. Wu, F. R. R. Moreira, M. Giovanetti, S. L. Li, J. G. de Jesus, F. J. Colón-González, H. R. Chamberlain, O. Pannell, N. Tejedor-Garavito, F. de Bruycker-Nogueira, A. A. Fabri, M. A. Mares-Guia, J. Xavier, A. E. Zarebski, A. Hamlet, M. A. M. Sallum, A. C. da Costa, E. R. Manuli, A. S. Levin, L. F. Mucci, R. M. Tubaki, R. M. T. de Menezes, J. T. de Deus, R. Spinola, L. Saad, E. G. Kallas, G. R. W. Wint, P. S. Peixoto, A. A. de Souza Santos, J. P. Messina, O. J. Brady, A. J. Tatem, M. A. Suchard, J. A. Mendez-Rico, A. Abreu, R. S. Aguiar, O. G. Pybus, G. Baele, P. Lemey, F. Iani, M. S. Cunha, Ana M. Bispo de Filippis, E. C. Sabino, N. R. Faria, Climate and land-use shape the spread of zoonotic yellow fever virus. medRxiv 22278983 (2022); 10.1101/2022.08.25.22278983.

[6] C. Possas, R. Lourenço-de-Oliveira, P. L. Tauil, F. de Paula Pinheiro, A. Pissinatti, R. V. da Cunha, M. Freire, R. M. Martins, A. Homma, Yellow fever outbreak in Brazil: the puzzle of rapid viral spread and challenges for immunisation. Mem. Inst. Oswaldo Cruz 113, e180278 (2018). 30427974 10.1590/0074-02760180278 PMC6135548

[7] J. I. Rosser, K. Nielsen-Saines, E. Saad, T. Fuller, Reemergence of yellow fever virus in southeastern Brazil, 2017-2018: What sparked the spread? PLoS Negl. Trop. Dis. 16, e0010133 (2022). 35130278 10.1371/journal.pntd.0010133 PMC8853510

[8] M. M. Gómez, F. V. S. de Abreu, A. A. C. dos Santos, I. S. de Mello, M. P. Santos, I. P. Ribeiro, A. Ferreira-de-Brito, R. M. de Miranda, M. G. de Castro, M. S. Ribeiro, R. da Costa Laterrière Junior, S. F. Aguiar, G. L. S. Meira, D. Antunes, P. H. M. Torres, D. Mir, A. C. P. Vicente, A. C. R. Guimarães, E. R. Caffarena, G. Bello, R. Lourenço-de-Oliveira, M. C. Bonaldo, Genomic and structural features of the yellow fever virus from the 2016-2017 Brazilian outbreak. J. Gen. Virol. 99, 536–548 (2018). 29469689 10.1099/jgv.0.001033

[9] P. de Oliveira Figueiredo, A. G. Stoffella-Dutra, G. Barbosa Costa, J. Silva de Oliveira, C. Dourado Amaral, J. Duarte Santos, K. L. Soares Rocha, J. P. Araújo Júnior, M. Lacerda Nogueira, M. A. Zazá Borges, A. Pereira Paglia, A. Desiree LaBeaud, J. Santos Abrahão, E. Geessien Kroon, D. Bretas de Oliveira, B. Paiva Drumond, G. de Souza Trindade, Re-emergence of yellow fever in Brazil during 2016-2019: Challenges, lessons learned, and perspectives. Viruses 12, 1233 (2020). 33143114 10.3390/v12111233 PMC7692154

[10] J. A. E. Santo, K. B. F. Zocratto, Febre amarela: cobertura vacinal na região metropolitana de Belo Horizonte. São Paulo: Revista Remecs. 4, 26–34 (2019).

[11] J. E. Staples, M. Gershman, M. Fischer, Centers for Disease Control and Prevention (CDC), Yellow fever vaccine: Recommendations of the Advisory Committee on Immunization Practices (ACIP). MMWR Recomm. Rep. 59, 1–27 (2010). 20671663

[12] O. M. Man, T. L. Fuller, J. I. Rosser, K. Nielsen-Saines, Re-emergence of arbovirus diseases in the State of Rio de Janeiro, Brazil: The role of simultaneous viral circulation between 2014 and 2019. One Health 15, 100427 (2022). 36277093 10.1016/j.onehlt.2022.100427 PMC9582545

[13] A. P. M. A. Cunha, M. Zeri, K. Deusdará Leal, L. Costa, L. A. Cuartas, J. A. Marengo, J. Tomasella, R. M. Vieira, A. A. Barbosa, C. Cunningham, J. V. Cal Garcia, E. Broedel, R. Alvalá, G. Ribeiro-Neto, Extreme drought events over Brazil from 2011 to 2019. Atmos. 10, 642 (2019).

[14] M. L. Childs, N. Nova, J. Colvin, E. A. Mordecai, Mosquito and primate ecology predict human risk of yellow fever virus spillover in Brazil. Philos. Trans. R Soc. Lond. B Biol. Sci. 374, 20180335 (2019). 31401964 10.1098/rstb.2018.0335 PMC6711306

[15] A. Estrada, R. Coates-Estrada, Tropical rain forest fragmentation and wild populations of primates at Los Tuxtlas, Mexico. Int. J. Primatol. 17, 759–783 (1996).

[16] N. C. C. de Azevedo Fernandes, J. M. Guerra, J. Díaz-Delgado, M. S. Cunha, L. delC Saad, S. D. Iglezias, R. A. Ressio, C. Dos Santos Cirqueira, C. T. Kanamura, I. P. Jesus, A. Y. Maeda, F. G. S. Vasami, J. de Carvalho, L. J. T. de Araújo, R. P. de Souza, J. S. Nogueira, R. M. F. Spinola, J. L. Catão-Dias, Differential yellow fever susceptibility in New World nonhuman primates, comparison with humans, and implications for surveillance. Emerg. Infect. Dis. 27, 47–56 (2021). 33350931 10.3201/eid2701.191220 PMC7774563

[17] J. C. Bicca-Marques, D. S. de Freitas, The role of monkeys, mosquitoes, and humans in the occurrence of a yellow fever outbreak in a fragmented landscape in South Brazil: Protecting howler monkeys is a matter of public health. Trop. Conserv. Sci. 3, 78–89 (2010).

[18] K. R. Amato, C. J. Yeoman, A. Kent, N. Righini, F. Carbonero, A. Estrada, H. Rex Gaskins, R. M. Stumpf, S. Yildirim, M. Torralba, M. Gillis, B. A. Wilson, K. E. Nelson, B. A. White, S. R. Leigh, Habitat degradation impacts black howler monkey (*Alouatta pigra*) gastrointestinal microbiomes. ISME J. 7, 1344–1353 (2013). 23486247 10.1038/ismej.2013.16 PMC3695285

[19] O. R. Causey, H. W. Kumm, H. W. Laemmert Jr., Dispersion of forest mosquitoes in Brazil: Further studies. Am. J. Trop. Med. Hyg. 30, 301–312 (1950). 15419408 10.4269/ajtmh.1950.s1-30.301

[20] R. W. Hagan, E. M. Didion, A. E. Rosselot, C. J. Holmes, S. C. Siler, A. J. Rosendale, J. M. Hendershot, K. S. B. Elliot, E. C. Jennings, G. A. Nine, P. L. Perez, A. E. Rizlallah, M. Watanabe, L. E. Romick-Rosendale, Y. Xiao, J. L. Rasgon, J. B. Benoit, Dehydration prompts increased activity and blood feeding by mosquitoes. Sci. Rep. 8, 6804 (2018). 29717151 10.1038/s41598-018-24893-z PMC5931509

[21] L. F. Chaves, L. C. Harrington, C. L. Keogh, A. M. Nguyen, U. D. Kitron, Blood feeding patterns of mosquitoes: Random or structured? Front. Zool. 7, 3 (2010). 20205866 10.1186/1742-9994-7-3 PMC2826349

[22] B. de Thoisy, N. I. O. Silva, L. Sacchetto, G. de Souza Trindade, B. P. Drumond, Spatial epidemiology of yellow fever: Identification of determinants of the 2016-2018 epidemics and at-risk areas in Brazil. PLoS Negl. Trop. Dis. 14, e0008691 (2020). 33001982 10.1371/journal.pntd.0008691 PMC7553304

[23] Intergovernmental Panel on Climate Change, *Climate Change Information for Regional Impact and for Risk Assessment* (Cambridge Univ. Press, Cambridge, 2023), pp. 1767–1926.

[24] M. A. Johansson, P. F. C. Vasconcelos, J. E. Staples, The whole iceberg: estimating the incidence of yellow fever virus infection from the number of severe cases. Trans. R. Soc. Trop. Med. Hyg. 108, 482–487 (2014). 24980556 10.1093/trstmh/tru092 PMC4632853

[25] D. Couto-Lima, Y. Madec, M. I. Bersot, S. S. Campos, M. de Albuquerque Motta, F. B. D. Santos, M. Vazeille, P. F. da Costa Vasconcelos, R. Lourenço-de-Oliveira, A.-B. Failloux, Potential risk of re-emergence of urban transmission of Yellow Fever virus in Brazil facilitated by competent *Aedes* populations. Sci. Rep. 7, 4848 (2017). 28687779 10.1038/s41598-017-05186-3 PMC5501812

[26] R. Lourenço-de-Oliveira, M. Vazeille, A. M. B. de Filippis, A.-B. Failloux, Oral susceptibility to yellow fever virus of *Aedes aegypti* from Brazil. Mem. Inst. Oswaldo Cruz 97, 437–439 (2002). 12048581 10.1590/s0074-02762002000300031

[27] F. V. S. de Abreu, I. P. Ribeiro, A. Ferreira-de-Brito, A. A. C. D. Santos, R. M. de Miranda, I. de Souza Bonelly, M. S. A. S. Neves, M. I. Bersot, T. P. D. Santos, M. Q. Gomes, J. L. da Silva, A. P. M. Romano, R. G. Carvalho, R. F. do Carmo Said, M. S. Ribeiro, R. da Costa Laperrière, E. O. L. Fonseca, A. Falqueto, C. Paupy, A.-B. Failloux, S. Moutailler, M. G. de Castro, M. M. Gómez, M. de Albuquerque Motta, M. C. Bonaldo, R. Lourenço-de-Oliveira, *Haemagogus leucocelaenus* and *Haemagogus janthinomys* are the primary vectors in the major yellow fever outbreak in Brazil, 2016-2018. Emerg. Microbes Infect. 8, 218–231 (2019). 30866775 10.1080/22221751.2019.1568180 PMC6455131

[28] M. S. Cunha, R. M. Tubaki, R. M. T. de Menezes, M. Pereira, G. S. Caleiro, E. Coelho, L. del Castillo Saad, N. C. C. de Azevedo Fernandes, J. M. Guerra, J. S. Nogueira, J. L. Summa, A. A. C. Coimbra, T. Zwarg, S. S. Witkin, L. F. Mucci, M. d. C. S. T. Timenetsky, E. C. Sabino, J. T. de Deus, Possible non-sylvatic transmission of yellow fever between non-human primates in São Paulo city, Brazil, 2017-2018. Sci. Rep. 10, 15751 (2020). 32978448 10.1038/s41598-020-72794-x PMC7519641

[29] M. S. Cunha, A. C. da Costa, N. C. C. de Azevedo Fernandes, J. M. Guerra, F. C. P. D. Santos, J. S. Nogueira, L. G. D’Agostino, S. V. Komninakis, S. S. Witkin, R. A. Ressio, A. Y. Maeda, F. G. S. Vasami, U. M. A. Kaigawa, L. S. de Azevedo, P. A. de Souza Facioli, F. L. L. Macedo, E. C. Sabino, É. Leal, R. P. de Souza, Epizootics due to Yellow Fever Virus in São Paulo State, Brazil: Viral dissemination to new areas (2016–2017). Sci. Rep. 9, 5474 (2019). 30940867 10.1038/s41598-019-41950-3 PMC6445104

[30] G. G. Pinheiro, M. N. Rocha, M. A. de Oliveira, L. A. Moreira, J. D. Andrade Filho, Detection of yellow fever virus in sylvatic mosquitoes during disease outbreaks of 2017-2018 in Minas Gerais State, Brazil. Insects 10, 136 (2019). 31083286 10.3390/insects10050136 PMC6572267

[31] J. Alencar, H. R. Gil-Santana, R. de Freitas Nunes de Oliveira, N. Dégallier, A. É. Guimarães, Natural breeding sites for *Haemagogus* mosquitoes (Diptera, Culicidae) in Brazil. Entomol. News 121, 393–396 (2010).

[32] J. Alencar, E. S. Lorosa, N. Dégallier, N. M. Serra-Freire, J. B. Pacheco, A. É. Guimarães, Feeding patterns of *Haemagogus janthinomys* (Diptera: Culicidae) in different regions of Brazil. J. Med. Entomol. 42, 981–985 (2005). 16465738

[33] J. Alencar, C. F. de Mello, F. Morone, H. G. Albuquerque, N. M. Serra-Freire, R. M. Gleiser, S. O. F. Silva, A. É. Guimarães, Distribution of *Haemagogus* and *Sabethes* species in relation to forest cover and climatic factors in the Chapada Dos Guimarães National Park, State of Mato Grosso, Brazil. J. Am. Mosq. Control Assoc. 34, 85–92 (2018). 31442164 10.2987/18-6739.1

[34] L. F. Mucci, R. P. C. Júnior, M. B. de Paula, S. A. S. Scandar, M. L. Pacchioni, A. Fernandes, C. A. Consales, Feeding habits of mosquitoes (Diptera: Culicidae) in an area of sylvatic transmission of yellow fever in the state of São Paulo, Brazil. J. Venom Anim. Toxins Incl. Trop. Dis. 21, 6 (2015). 25810711 10.1186/s40409-015-0005-z PMC4373060

[35] Y. Liu, J. Rocklöv, What is the reproductive number of yellow fever? J. Travel Med. 27, taaa156 (2020). 32889541 10.1093/jtm/taaa156 PMC7649379

[36] R. K. Plowright, A. N. Ahmed, T. Coulson, T. W. Crowther, I. Ejotre, C. L. Faust, W. F. Frick, P. J. Hudson, T. Kingston, P. O. Nameer, M. T. O’Mara, A. J. Peel, H. Possingham, O. Razgour, D. A. M. Reeder, M. Ruiz-Aravena, N. B. Simmons, P. N. Srinivas, G. M. Tabor, I. Tanshi, I. G. Thompson, A. T. Vanak, N. M. Vora, C. E. Willison, A. T. H. Keeley, Ecological countermeasures to prevent pathogen spillover and subsequent pandemics. Nat. Commun. 15, 2577 (2024). 38531842 10.1038/s41467-024-46151-9 PMC10965931

[37] A. Aliaga-Samanez, R. Real, M. Segura, C. Marfil-Daza, J. Olivero, Yellow fever surveillance suggests zoonotic and anthroponotic emergent potential. Commun. Biol. 5, 530 (2022). 35654842 10.1038/s42003-022-03492-9 PMC9163115

[38] T. Mueller, K. A. Olson, G. Dressler, P. Leimgruber, T. K. Fuller, C. Nicolson, A. J. Novaro, M. J. Bolgeri, D. Wattles, S. DeStefano, J. M. Calabrese, W. F. Fagan, How landscape dynamics link individual- to population-level movement patterns: A multispecies comparison of ungulate relocation data. Glob. Ecol. Biogeogr. 20, 683–694 (2011).

[39] V. B. Fortes, J. C. Bicca-Marques, B. Urbani, V. A. Fernández, T. da Silva Pereira, “Ranging behavior and spatial cognition of howler monkeys” in *Howler Monkeys: Behavior, Ecology, and Conservation*, M. M. Kowalewski, P. A. Garber, L. Cortés-Ortiz, B. Urbani, D. Youlatos, Eds. (Springer New York, New York, NY, 2015), pp. 219–255.

[40] G. Pozo-Montuy, J. C. Serio-Silva, Movement and resource use by a group of *Alouatta pigra* in a forest fragment in Balancáne, México. Primates 48, 102–107 (2007). 17136475 10.1007/s10329-006-0026-x

[41] I. J. Amanna, M. K. Slifka, Questions regarding the safety and duration of immunity following live yellow fever vaccination. Expert Rev. Vaccines 15, 1519–1533 (2016). 27267203 10.1080/14760584.2016.1198259 PMC5171234

[42] A. J. McMichael, Extreme weather events and infectious disease outbreaks. Virulence 6, 543–547 (2015). 26168924 10.4161/21505594.2014.975022 PMC4720230

[43] J. Shaman, J. F. Day, M. Stieglitz, Drought-induced amplification and epidemic transmission of West Nile virus in southern Florida. J. Med. Entomol. 42, 134–141 (2005). 15799522 10.1093/jmedent/42.2.134

[44] G. E. C. Charnley, I. Kelman, N. Green, W. Hinsley, K. A. M. Gaythorpe, K. A. Murray, Exploring relationships between drought and epidemic cholera in Africa using generalised linear models. BMC Infect. Dis. 21, 1177 (2021). 34809609 10.1186/s12879-021-06856-4 PMC8609751

[45] C. Stanke, M. Kerac, C. Prudhomme, J. Medlock, V. Murray, Health effects of drought: A systematic review of the evidence. PLoS Curr. 5, 10.1371/currents.dis.7a2cee9e980f91ad7697b570bcc4b00 (2013). 10.1371/currents.dis.7a2cee9e980f91ad7697b570bcc4b004 PMC3682759 23787891

[46] S. Mukherjee, A. Mishra, K. E. Trenberth, Climate change and drought: A perspective on drought indices. Curr. Clim. Change Rep. 4, 145–163 (2018).

[47] J. H. Huber, M. L. Childs, J. M. Caldwell, E. A. Mordecai, Seasonal temperature variation influences climate suitability for dengue, chikungunya, and Zika transmission. PLoS Negl. Trop. Dis. 12, e0006451 (2018). 29746468 10.1371/journal.pntd.0006451 PMC5963813

[48] V. Eyring, S. Bony, G. A. Meehl, C. A. Senior, B. Stevens, R. J. Stouffer, K. E. Taylor, Overview of the Coupled Model Intercomparison Project Phase 6 (CMIP6) experimental design and organization. Geosci. Model Dev. 9, 1937–1958 (2016).

[49] M. Amaku, F. A. B. Coutinho, E. Massad, Why dengue and yellow fever coexist in some areas of the world and not in others? Biosystems 106, 111–120 (2011). 21839800 10.1016/j.biosystems.2011.07.004

[50] T. W. Scott, P. H. Amerasinghe, A. C. Morrison, L. H. Lorenz, G. G. Clark, D. Strickman, P. Kittayapong, J. D. Edman, Longitudinal studies of *Aedes aegypti* (Diptera: Culicidae) in Thailand and Puerto Rico: blood feeding frequency. J. Med. Entomol. 37, 89–101 (2000). 15218911 10.1603/0022-2585-37.1.89

[51] M. A. Johansson, N. Arana-Vizcarrondo, B. J. Biggerstaff, N. Gallagher, N. Marano, J. E. Staples, Assessing the risk of international spread of yellow fever virus: A mathematical analysis of an urban outbreak in Asuncion, 2008. Am. J. Trop. Med. Hyg. 86, 349–358 (2012). 22302873 10.4269/ajtmh.2012.11-0432 PMC3269406

[52] E. Hindle, The transmission of yellow fever. Lancet 216, 835–842 (1930).

[53] M. Bates, M. Roca-Garcia, Laboratory studies of the *Saimiri*-*Haemagogus* cycle of jungle yellow fever. Am. J. Trop. Med. Hyg. s1-25, 203–216 (1945).

[54] M. Bates, M. Roca-García, Experiments with various Colombian marsupials and primates in laboratory cycles of yellow fever. Am. J. Trop. Med. Hyg. 26, 437–453 (1946). 20996628 10.4269/ajtmh.1946.s1-26.437

[55] J. H. Bauer, N. P. Hudson, The incubation period of yellow fever in the mosquito. J. Exp. Med. 48, 147–153 (1928). 19869467 10.1084/jem.48.1.147 PMC2131459

[56] T. P. Monath, Yellow fever: An update. Lancet Infect. Dis. 1, 11–20 (2001). 11871403 10.1016/S1473-3099(01)00016-0

[57] M. A. Johansson, N. Arana-Vizcarrondo, B. J. Biggerstaff, J. E. Staples, Incubation periods of yellow fever virus. Am. J. Trop. Med. Hyg. 83, 183–188 (2010). 20595499 10.4269/ajtmh.2010.09-0782 PMC2912597

[58] M. Bates, M. Roca-García, The development of the virus of yellow fever in haemagogus mosquitoes. Am. J. Trop. Med. Hyg. 26, 585–605 (1946). 21003267 10.4269/ajtmh.1946.s1-26.585

[59] B. Mondet, Condições de sobrevivência em laboratório de *Haemagogus janthinomys* Dyar, 1921 (Diptera: Culicidae). Rev. Soc. Bras. Med. Trop. 30, 11–14 (1997). 9026827 10.1590/s0037-86821997000100003

[60] N. Dégallier, G. C. SÁ Filho, H. A. O. Monteiro, F. C. Castro, O. Vaz da Silva, R. C. F. Brandão, M. Moyses, A. P. A. T. da Rosa, Release–recapture experiments with canopy mosquitoes in the genera *Haemagogus* and *Sabeihes* (Diptera: Culicidae) in Brazilian Amazonia. J. Med. Entomol. 35, 931–936 (1998). 9835682 10.1093/jmedent/35.6.931

[61] D. Goindin, C. Delannay, C. Ramdini, J. Gustave, F. Fouque, Parity and longevity of *Aedes aegypti* according to temperatures in controlled conditions and consequences on dengue transmission risks. PLOS ONE 10, e0135489 (2015). 26258684 10.1371/journal.pone.0135489 PMC4530937

[62] J. Froehlich, R. W. Thorington, J. Otis, The demography of howler monkeys (*Alouatta palliata*) on Barro Colorado Island, Panamá. Int. J. Primatol. 2, 207–236 (1981).

[63] S. Tardif, C. Ross, D. Smucny, “Building marmoset babies: Trade-offs and cutting bait” in *Building Babies: Primate Development in Proximate and Ultimate Perspective*, K. Clancy, K. Hinde, J. Rutherford, Eds. (Springer, New York, 2013), pp. 169–183.

[64] L. Wachtman, K. Mansfield, “Viral diseases of nonhuman primates” in *Nonhuman Primates in Biomedical Research: Diseases*, C. R. Abee, K. Mansfield, S. D. Tardif, T. Morris, Eds. (Academic Press, San Diego, ed. 2, 2012), pp. 1–104.

[65] E. S. Moreno, I. Agostini, I. Holzmann, M. S. di Bitetti, L. I. Oklander, M. M. Kowalewski, P. M. Beldomenico, S. Goenaga, M. Martínez, E. Lestani, A. L. J. Desbiez, P. Miller, Yellow fever impact on brown howler monkeys (*Alouatta guariba clamitans*) in Argentina: A metamodelling approach based on population viability analysis and epidemiological dynamics. Mem. Inst. Oswaldo Cruz 110, 865–876 (2015). 26517499 10.1590/0074-02760150075 PMC4660615

[66] H. W. Laemmert Jr., Susceptibility of marmosets to different strains of yellow fever virus. Am. J. Trop. Med. Hyg. s1-24, 71–81 (1944).

[67] P. F. da Costa Vasconcelos, Febre amarela. Rev. Soc. Bras. Med. Trop. 36, 275–293 (2003). 12806465 10.1590/s0037-86822003000200012

[68] WHO, Disease Outbreak News Yellow fever - Brazil (2019), www.who.int/emergencies/disease-outbreak-news/item/18-april-2019-yellow-fever-brazil-en.

[69] State Secretariat of Health of Minas Gerais, “Febre Amarela” (Secretaria de Estado de Saúde de Minas Gerais, Belo Horizonte, 2026); www.saude.mg.gov.br/febreamarela/.

