# Supplementary material for: Drought dynamics explain once in a century yellow fever virus outbreak in Brazil with implications for climate change
Source: Sci Adv. 2026 Aug 14;12(33):eadz6832. doi: 10.1126/sciadv.adz6832 (PMC13475486; doi:10.1126/sciadv.adz6832)
Supplement: Supplementary file 1 [file sciadv.adz6832_sm.pdf]

Science Advances
AAAS

Supplementary Materials for

Drought dynamics explain once in a century yellow fever virus outbreak in Brazil with implications for climate change

Jamie M. Caldwell *et al.*

Corresponding author: Jamie M. Caldwell, jamie.caldwell@princeton.edu; Joelle I. Rosser, jrosser@stanford.edu

Sci. Adv. **12**, eadz6832 (2026)
DOI: 10.1126/sciadv.adz6832

This PDF file includes:

Sensitivity analyses
Analysis removing *Ae. aegypti*
Figs. S1 to S3
Tables S1 to S3

Sensitivity analyses

The model results were relatively robust to highly uncertain parameters (figure S1, table S2). Increasing and decreasing the YFV-induced mortality rate in monkeys decreased and increased incidence, respectively. In other words, the highest mortality rate resulted in lower incidence, presumably because the monkey population was dying too quickly to amplify transmission. The model was most sensitive to increasing the probability of mosquito infection with YFV: in non-human primates, it resulted in a slightly earlier and higher peak in incidence during the first wave of transmission with an earlier start to the second wave of transmission, while in humans, incidence was substantially higher during both peaks and the second wave occurred earlier than observed. Increasing and decreasing movement rates of forest species increased and decreased incidence, respectively, but also affected the timing of transmission (primarily for low movement rates). The average correlation across all sensitivity analysis simulations was lower than the full model. Additionally, we found the most important model parameter was seasonally driven based on a global sensitivity analysis (figures S2, S3).

Transmission dynamics without *Aedes aegypti*

Since both observational data and the model results suggested *Aedes aegypti* were unimportant for this outbreak, we tested the fit of the full model while setting the *Ae. aegypti* classes to zero. The resulting model reproduced the general dynamics of the outbreak but showed a modest reduction in fit for most metrics compared with the full model (table S3).

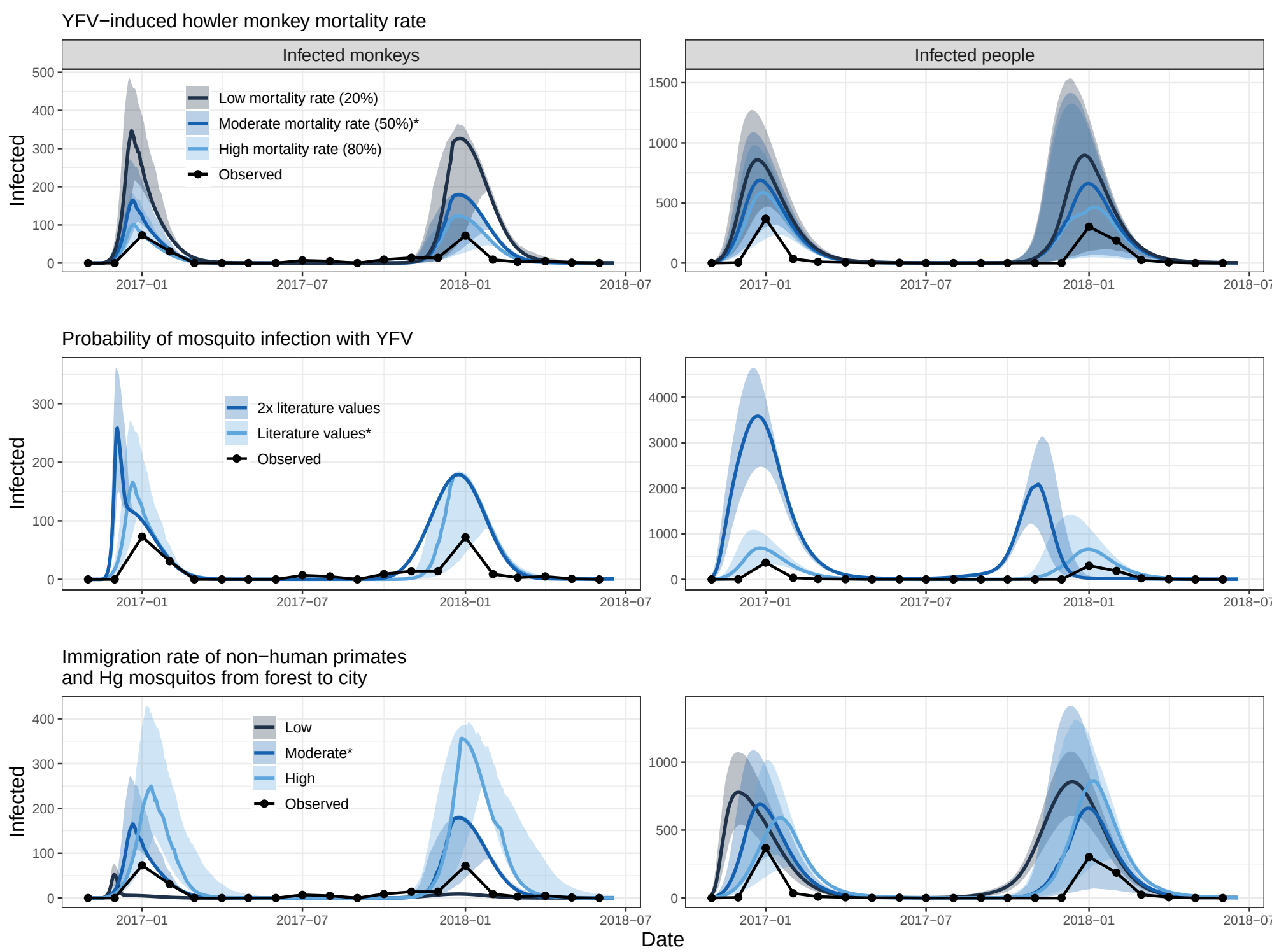

Figure S1: **Model results are relatively robust to uncertain parameter values.** Infected monkeys (left) and infected people (right). Comparison of model simulations using low and high YFV-induced monkey mortality rates (top), high probability of mosquito infection with YFV (middle), and low and high movement rates for monkeys, marmosets, and *Haemagogus* mosquitoes (bottom). We indicated the values used in the full model with an asterisk and show observations (black points connected with lines) for comparison.

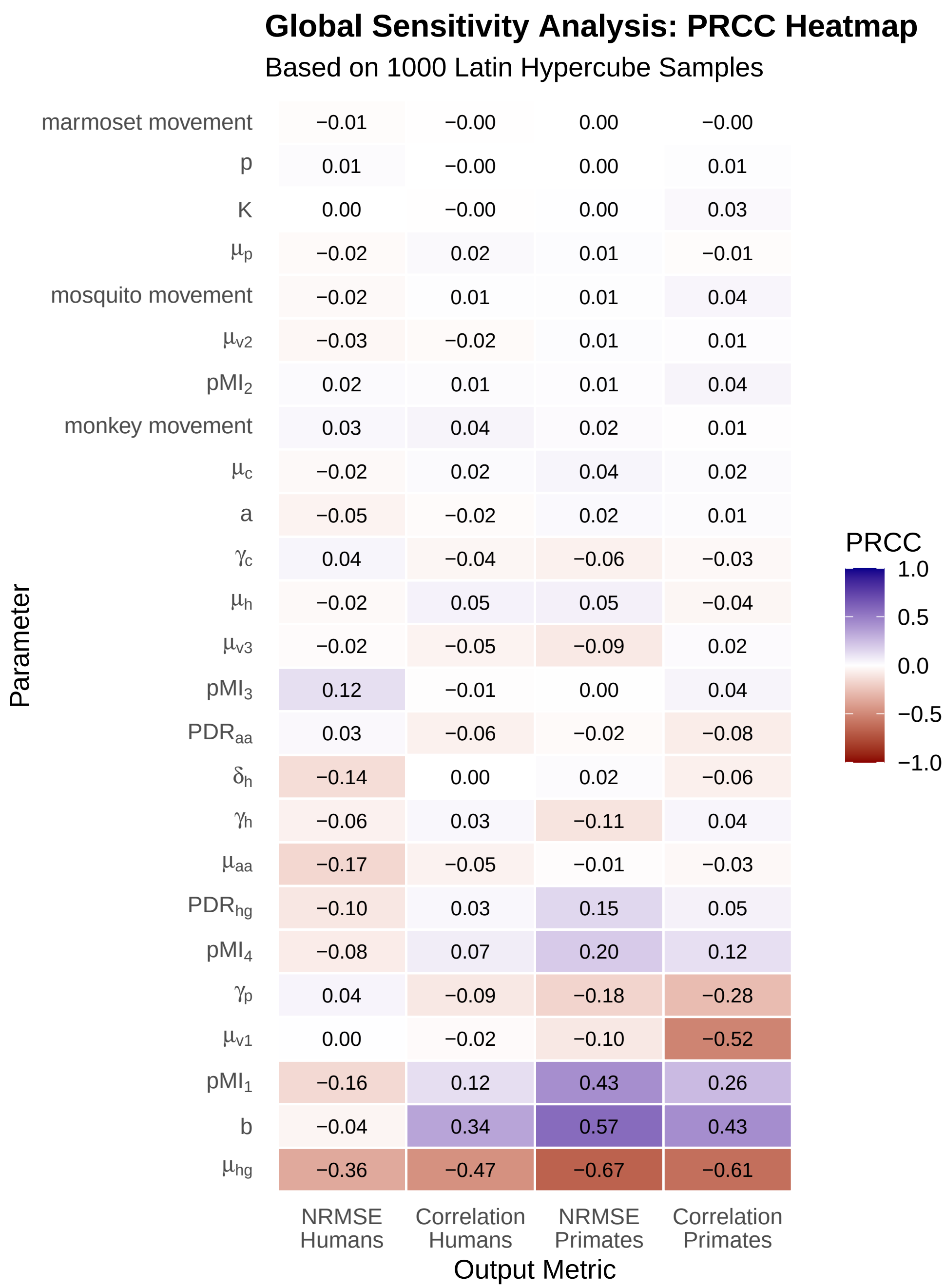

Figure S2: **Partial Rank Correlation Coefficient (PRCC) heatmap for global sensitivity analysis of model parameters.** Values represent PRCCs between 26 input parameters and four model output metrics: correlation and normalized RMSE (NRMSE) for both human and non-human primate cases. Strongest correlations are observed for seasonal mosquito mortality (μ_{hg}), transmission rate (b), and primate recovery rate (γ_p). Parameters with high PRCC values (< 0.4) and $p < 0.05$ are considered highly influential. Negative PRCC values indicate inverse relationships. Results are based on 1,000 Latin Hypercube Samples.

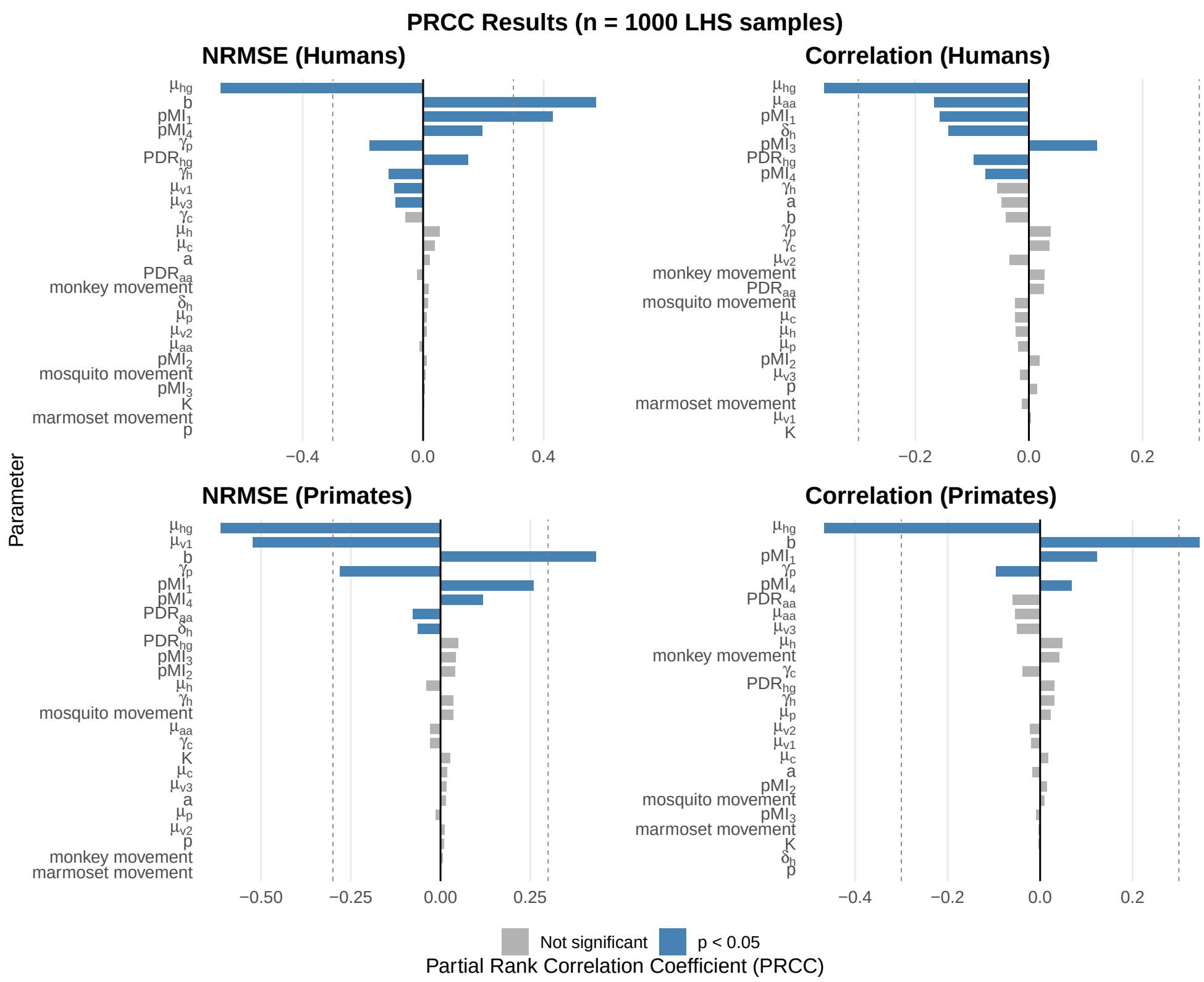

Figure S3: **Partial Rank Correlation Coefficient (PRCC) values between model parameters and output metrics.** Each row represents a model parameter; columns represent PRCC values for the four output metrics: human incidence correlation, human incidence NRMSE, primate incidence correlation, and primate incidence NRMSE. Statistically significant correlations ($p < 0.05$) are shown in blue bars. Parameters include both seasonal and non-seasonal components.

Table S1: **Distribution functions for generating the 500 initial condition values.** Note that the *Haemagogus* mosquito and marmoset populations are proportions of the *Aedes aegypti* and howler monkey populations; thus, the initial conditions for these compartments varied across the simulations accordingly producing variation in initial conditions for five compartments.

Compartment	Distribution function
S_{aa}	˜Normal(300000, 100000)
S_p	˜Uniform(35000, 10000)
R_p	˜Normal(0.7, 0.1)

Table S2: **Correlation (and $p - value$) and normalized root mean squared error between model predictions and observations across nested seasonally-driven models.** Modeled human cases were adjusted by a one month lead time. NHP: non-human primates. Note that the 'No seasonality' model still includes a seasonal mosquito carrying capacity. We included the full model in the table for comparison.

Model	Human cases	NHP cases	Average correlation; NRMSE
Full model	0.90 ($p < 0.001$); 4	0.90 ($p < 0.001$); 3	0.90; 3.5
Low YFV-monkey induced mortality rate	0.87 ($p < 0.001$); 6	0.91 ($p < 0.001$); 7	0.89; 6.5
High YFV-monkey induced mortality rate	0.88 ($p < 0.001$); 3	0.89 ($p < 0.001$); 2	0.89; 2.5
High probability of mosquito infection with YFV	0.42 ($p < 0.05$); 26	0.60 ($p < 0.05$); 5	0.51; 15.5
Low movement rate of forest species	0.54 ($p < 0.01$); 7	0.09 ($p < 0.05$); 2	0.32; 4.5
High movement rate of forest species	0.82 ($p < 0.001$); 5	0.88 ($p < 0.001$); 8	0.85; 6.5

Table S3: **Comparison of model performance with and without *Aedes aegypti* vector classes.** Correlation and normalized root mean square error (NRMSE) metrics for both human and non-human primate incidence. The full model (including *Ae. aegypti*) achieved higher correlation for both hosts, while the model excluding *Ae. aegypti* showed a slight improvement in NRMSE for humans but a notably poorer fit for primates. These results suggest that *Ae. aegypti* were not major drivers of transmission during the outbreak but may have had a minor influence on overall model dynamics, particularly in non-human primates.

Model	Metric	Human cases	Primate cases
Full model	Correlation	0.90	0.90
Model without *Ae. aegypti*	Correlation	0.82	0.64
Full model	NRMSE	4	3
Model without *Ae. aegypti*	NRMSE	2	6
